# Hydrogen Sulfide Attenuated Angiotensin II-Induced Sympathetic Excitation in Offspring of Renovascular Hypertensive Rats

**DOI:** 10.3389/fphar.2020.565726

**Published:** 2020-09-11

**Authors:** Xiaohong Feng, Qi Guo, Hongmei Xue, Xiaocui Duan, Sheng Jin, Yuming Wu

**Affiliations:** ^1^Department of Laboratory Diagnostics, Hebei Medical University, Shijiazhuang, China; ^2^Experimental Center for Teaching, Hebei Medical University, Shijiazhuang, China; ^3^Department of Physiology, Hebei Medical University, Shijiazhuang, China; ^4^Hebei Key Laboratory of Animal Science, Hebei Medical University, Shijiazhuang, China; ^5^Hebei Collaborative Innovation Center for Cardio-Cerebrovascular Disease, Shijiazhuang, China; ^6^Key Laboratory of Vascular Medicine of Hebei Province, Shijiazhuang, China

**Keywords:** sympathetic excitation, hypertension, nucleus tractus solitarii, angiotensin II, fetal programming

## Abstract

**Objective:**

Numerous findings have demonstrated a strong association between parental health during pregnancy and cardiovascular disease in adult offspring. This study investigated whether sensitivity to angiotensin II (Ang II) is enhanced in offspring of renovascular hypertensive animals and whether hydrogen sulfide (H_2_S) can attenuate the increased response to Ang II in offspring.

**Method:**

The systolic blood pressure (SBP) was measured by non-invasive tail-cuff plethysmograpy every two weeks in all offspring from 8 to 16 weeks. After intracerebroventricular microinjection of Ang II in the offspring, blood pressure, heart rate (HR), and renal sympathetic nerve activity (RSNA) were recorded to test the response to Ang II in the offspring. Western blot analysis was used to examine the protein expression of AT1R, AT1R-associated protein (ATRAP), Nox2, p67^phox^, and nitrotyrosine in the nucleus tractus solitarii (NTS).

**Results:**

The SBP in the offspring of hypertensive rats were significantly higher than that in control group, and the above effects were significantly improved by prenatal or postnatal administration of H_2_S. Intralateroventricular microinjection of Ang II induced greater sympathetic responses in offspring of hypertensive rats than control group. The expression of AT1R and oxidative stress-related protein was increased, whereas that of ATRAP was decreased in the NTS in offspring of hypertensive rats. Exogenous administration of H_2_S prenatally or postnatally improved the above effects.

**Conclusion:**

Prenatal or postnatal administration of H_2_S attenuated AngII-induced sympathetic excitation in offspring of hypertensive rats, which may occur by modulating the balance between AT1R and ATRAP and downregulating oxidative stress-related protein expression in the NTS.

## Introduction

Increasing evidence has shown that an adverse intrauterine environment, such as in mothers with hypertension and diabetes, increases the risk of cardiovascular diseases in adult offspring (Barker and Osmond, 1986; Kobori et al., 2007; Samuelsson et al., 2013). For example, in women with preeclampsia in pregnancy, their children tend to have cardiovascular disease later in life (Bokslag et al., 2016). A similar study reported that children of women with preeclampsia showed increased risk of stroke in adulthood (Kajantie et al., 2009). In pregnant women with diabetes, the probability of cardiovascular diseases was increased in their adult offspring (Ma et al., 2015). Moreover, nicotine exposure during pregnancy leads to increased risk of hypertension and metabolic disorders in adult offspring (Xiao et al., 2008; Somm et al., 2009). Animal experiments have shown that uteroplacental insufficiency, protein restriction, chronic secondary hypertension, or glucocorticoid treatment during pregnancy leads to hypertension in offspring (Liang et al., 2013). However, early intervention can prevent the development of adult hypertension (Koeners et al., 2010; Koeners et al., 2011).

Renovascular hypertension (RVH) induced by partial renal artery stenosis could lead to persistent high blood pressure (BP) and increased angiotensin II (AngII) level (Higashi et al., 2002; Takahashi and Smithies, 2004). AngII, the major effector peptide of the renin-angiotensin-aldosterone system (RAAS), increased the production of nicotinamide adenine dinucleotide phosphate (NADPH) oxidase, especially superoxide anion (O^2 -^), in a rat model of renovascular hypertension (Oliveira-Sales et al., 2009) *via* activating AngII type I receptor (AT1R). AT1R-associated protein (ATRAP) specifically combines with the carboxyl-terminal domain of AT1R (Lopez-Ilasaca et al., 2003). In vitro study showed that ATRAP suppressed AngII-mediated pathological responses in cardiovascular cells by promoting AT1R internalization (Tamura et al., 2013), and ATRAP also suppressed the role of AT1R in the pathological process of hypertension (Dejima et al., 2011).

The nucleus tractus solitarius (NTS) is the primary site of peripheral afferent impulses to the central nervous system, receiving cardiovascular afferent signals after preliminary integration and transfer to nuclei that regulate the circulatory variables. Previous studies have shown that AT1R are widely distributed in the central nervous system and play an important role in the development of hypertension (Xu et al., 1998). Also, AT1R is abundantly expressed in the NTS (Hirooka, 2011). Abnormalities of the RAAS in the NTS is involved in the pathophysiological process of hypertension (Matsumura et al., 1998; Diz et al., 2011). Therefore, RAAS expressed in the NTS could play a key role in regulating BP stability, sympathetic outflow, and baroreceptor reflex control of cardiovascular function (Hirooka et al., 2003; Zanutto et al., 2010; Zanutto et al., 2011).

Hydrogen sulfide (H_2_S) is considered the third gas signal molecule after NO and CO and is involved in many physiological and pathophysiological processes in the cardiovascular system (Wang, 2002). Physiological concentrations of H_2_S can relax blood vessels, inhibit oxidative stress, decrease myocardial injury, attenuate inflammation (Polhemus and Lefer, 2014), and reduce BP both in normotensive and hypertensive rats (Ford et al., 2013; Ahmad et al., 2014). We found that the plasma level of H_2_S was significantly decreased in the model of RVH (Xiao et al., 2018), which leads to oxidative stress. However, exogenous administration of NaHS could rapidly increase the H_2_S level in renovascular hypertensive rats (Xiao et al., 2016). By inhibiting AT1 receptor and oxidative stress-related proteins, H_2_S could lower BP in RVH rats (Xue et al., 2015; Guo et al., 2017).

Maternal environment plays an important role in maintaining normal physiological function after birth. Therefore, preventive measures are needed to prevent and cure cardiovascular disease in the perinatal period (Marco et al., 2012). In this study, we examined whether prenatal and postnatal treatment with H_2_S could attenuate AngII-induced sympathetic excitation in hypertensive offspring by decreasing the level of AT1 receptor and increasing that of ATRAP.

## Methods

### Preparation of Parental Hypertensive Model

Seven-week male and female Sprague-Dawley rats (180–220 g) were obtained from the Animal Research Center of Hebei Medical University and kept in ordinary cages at 22 ± 2°C with 12-h dark/light cycles (lights on 6:00) with food and water adlibitum. All animal procedures complied with the Animal Management Rule of the Ministry of Health, People’s Republic of China (documentation no. 55, 2001) and the Care and Use of Laboratory Animals published by the US National Institutes of Health (NIH publication no. 85-23, revised in 1996) and were approved by the Animal Care Committee of Hebei Medical University. Seven-week-old male and female Sprague-Dawley (SD) rats, weighting 160–180 g, were anesthetized with pentobarbital sodium (50 mg/kg, i.p.). The absence of a withdrawal response to a paw pinch was used to confirm adequate anesthesia. The left kidney was exposed *via* laparotomy, and the left renal artery was carefully separated from the left renal vein and connective tissue. Then, the left renal artery was clipped with a rigid U-shaped solid clip. The stainless-steel clip with an opening of 0.25 mm (Aowang Medical Instrument Company, Shanghai) was used to clamp the left renal artery to induce stenosis, thus resulting in hypertension. Thereafter, animals were given a subcutaneous injection of penicillin G (30,000 U/100 g) and meloxicam (1 mg/kg).

### Mating and Groups

The parental hypertensive rats were trained to become accustomed to the procedures for systolic blood pressure (SBP) measurement. Each rat was placed on a heating pad for 15–20 min to promote vasodilatation of the tail artery before measurement of SBP. SBP was measured in conscious rats each week after surgery for 5 weeks by tail-cuff plethysmography (Chengdu Instrument Factory, Sichuan, China). While SBP in RVH rats was up to 150 mmHg in 5 weeks after surgery, we selected 12 pairs of male and female RVH rats to mate and produce offspring. There were four groups in the experiment: The control group was offspring of SD rats, F_1N_F_2N_ rats were offspring of RVH rats, F_1H_F_2N_ rats were maternal RVH rats treated with NaHS (H_2_S donor, 56 μmol/kg per day, i.p.) during pregnancy and lactation and offspring without NaHS, and F_1N_F_2H_ rats were maternal RVH rats treated without NaHS during pregnancy and lactation and offspring with NaHS after weaning. On the first day of pregnancy, each mother received NaHS treatment or not. Each female rat was separately mated overnight. The day of pregnancy was defined as the day when spermatozoa were found in a vaginal smear. In the offspring in the above groups, SBP was measured from 8 to 16 weeks.

### Blood Pressure Measurement in Conscious Offspring

SBP was measured non-invasively by tail-cuff plethysmography in conscious offspring from 8 to 16 weeks, every 2 weeks. The rats were trained to become accustomed to the procedures for blood pressure measurement before the experiments. Each rat was placed on a heating pad for 15–20 min to promote vasodilatation of the tail artery prior to measurement of SBP. BP measurement was always carried out between 9:00 and 12:00, and the data were obtained with the average of three to four times.

### Implanting Cannula Into the Lateral Ventricle of Offspring

After anesthetization with pentobarbital sodium (50 mg/kg, i.p.), 15 weeks offspring were placed in a prone position, and the head of the animal was fixed on a stereotaxic frame. The skin of the skull bone was removed *via* a midline incision to expose the bregma. The stereotaxic coordinates used were the following (in mm): caudal to bregma, 0.8; lateral to midline, 1.5; and ventral to dura, 3.5 to 3.8. Offspring were mounted on a stereotaxic apparatus and implanted with a lateral cerebroventricular cannula. At 1 week after surgery, rats were anesthetized with pentobarbital sodium, and then Ang II (10 and 30 ng, 5 μL) was injected in the lateral ventricle *via* the cannula connected to a microsyringe with a polyethylene tube. BP, HR, and RSNA were recorded simultaneously by using PowerLab software (AD Instruments, Australia).

### Recording of BP, HR, and RSNA

Pentobarbital sodium (50 mg/kg, i.p.) was used to anesthetize rats, and then the PE tube was cannulated into their trachea for ventilation. A thermostatic bed was used to maintain body temperature in the range of 37–38℃. The left femoral artery was cannulated and connected with a pressure transducer to record arterial BP (ABP). The HR was measured along with BP. The left kidney was exposed *via* a retroperitoneal approach. One branch of the left renal sympathetic nerve around the renal vessels was isolated from the surrounding tissue carefully. To eliminate the nerve’s afferent activity, it was cutted distally. The nerve was placed on a bipolar platinum electrode for potential recording and immersed in warm (37°C) mineral oil. The nerve signal was amplified (**20**,**000**–**30**,**000**) and band-pass–filtered (100–3,000 Hz) by an alternating current amplifier (model P511; Grass Instruments, West Warwick, RI, USA). The pressure and nerve discharge signal were amplifier-fed into a PowerLab 15T data acquisition system (AD Instruments, QUAD, Bridge, Australia) to record ABP and RSNA simultaneously. A customized computer program was used to integrate the RSNA; the integrating time was 0.16 s before, and 5 min after, rats were euthanized by an overdose of sodium pentobarbital (200 mg/kg, i.v.) to suppress RSNA completely to obtain background electrical noise. The electrical noise level measured with these two methods was similar and was subtracted from the integrated RSNA values, and the percentage change in RSNA from baseline was calculated.

### Collecting NTS Tissue

At the end of the experiment, brains were dissected, and fresh brainstem tissue was transected coronally at the level of the NTS, which was identified as a distinct translucent structure in the dorsomedial medulla. Histology verification referred to Paxinos and Watson′s coordinates. At the level of the area postrema, a 250-μm thick piece of NTS was punched out bilaterally by using fine-tip forceps under a binocular microscope. The tissues were immersed in artificial cerebrospinal fluid at 0°C in the whole process. The NTS tissues from each rat were rapidly removed and placed in liquid nitrogen, then stored at −80°C.

### Western Blot Detection of Protein

NTS tissues were added with pre-cooled lysate and then cracked by ultrasonography. The specimens were statically placed on ice for 30 min to be fully cracked and then centrifuged at 12,000 rpm for 15 min at 4°CC. Supernatant was collected for protein assay. The Bradford assay (Generay Biotechnology, Shanghai) was used to determine the concentration of protein in tissue. An amount of 5 μg of protein was loaded in each lane. The protein was separated by electrophoresis, transferred onto polyvinylidene fluoride membranes, which were blocked with 0.1% Tween-20 tris-buffered saline containing 10% nonfat milk for 1 h at room temperature, and then incubated overnight at 4°C with the rabbit polyclonal antibodies anti-AT1R (1:800; ab18801, Abcam, USA), anti-ATRAP (1:500; sc-134652, Santa Cruz Biotechnology), anti-Nox2 (1:1,000; Abcam), anti-p67phox (1:1,000; EPITOMICS, USA), and anti-nitrotyrosine (1:1,000; Millipore), followed by the peroxidase-conjugated goat anti-rabbit secondary antibody (1:2,000; SA00001-2, Proteintech Biotechnology). Western blotting reagents (Millipore Corp., Billerica, MA) were used to detect the signal and blots were exposed to X-ray film for densitometric analysis.

### Statistical Analysis

All data are expressed as means ± SE. Repeated measures analysis of variance was tested to compare diﬀerences between groups at several times. One-way ANOVA was applied to compare diﬀerences between groups. Student-Newman-Keuls test and Bonferroni correction were used for analysis at one time. Differences were considered statistically significant at *P* < 0.05.

## Results

### SBP in Conscious Offspring

We measured SBP every 2 weeks from 8 to 16 weeks. Firstly for SBP, the interaction between times and groups had significant eﬀect on subjects (*P* < 0.05). Times had no significant eﬀect (*P* > 0.05) on subjects while groups had (*P* < 0.05). The SBP in male offspring of renovascular hypertensive dams significantly higher compared with that in control group from 8 to 16 weeks. And the above effects were significantly improved by exogenous administration with H_2_S. When we treated the hypertensive mother with H_2_S during pregnancy and lactation, the offspring exhibited a persistence decrease in blood pressure compared with hypertensive offspring. The blood pressure was decreased gradually in the offspring which treated with H_2_S after weaning. In female offspring of renovascular hypertensive dams, the SBP significantly elevated when compared with control from 8 to 16 weeks. In addition, the SBP in male offspring of renovascular hypertensive dams was higher than that in female offspring, while there was no sex different in normal rat (**Table 1**).

**Table 1:** Time courses of changes in systolic blood pressure (SBP) in four different groups of male and female offspring.

	Groups	8W	10W	12W	14W	16W
Male	F_1N_F_2N_ F_1H_F_2N_ F_1N_F_2H_ Control	131.4 ± 2.6* 120.8 ± 2.7**^#^** 126.6 ± 2.1 111.6 ± 1.8	133 ± 1.9* 120.2 ± 1.2**^#^** 124.3 ± 1.8**^#^** 112.3 ± 1.5	132.4 ± 1.9* 119.1 ± 0.9**^#^** 124.6 ± 1.2**^#^** 111 ± 1.8	133.2 ± 1.9* 120.2 ± 1.6**^#^** 118.8 ± 1.2**^#^** 112.2 ± 1.4	135 ± 2.8* 117.5 ± 2.3**^#^** 114.8 ± 1.2**^#^** 113.1 ± 1.6
Female	F_1N_F_2N_ F_1H_F_2N_ F_1N_F_2H_ Control	123.4 ± 1.3*^♦^ 115 ± 2**^#^** 124.9 ± 1.8 107.6 ± 2	122.7 ± 1.4*^♦^ 119.9 ± 1.5 122.5 ± 2.7 108.2 ± 1.3	124.2 ± 1.7*^♦^ 117.2 ± 2.4 120.7 ± 2.2 109.2 ± 2.1	119.9 ± 2.6*^♦^ 117.8 ± 1 115.9 ± 2.8 108.8 ± 2.2	115.9 ± 0.6*^♦^ 115.4 ± 1.3 108 ± 2.8**^#^** 108.7 ± 2.3

Date are showed as mean ± SE (n = 40). *P < 0.05 compared with control, ^#^P < 0.05 compared with F_1N_F_2N,_ and ^♦^P < 0.05 compared with male F_1N_F_2N_.

### Sympathetic Response to AngⅡ in Rat Offspring

#### Sympathetic Response to Low-Dose Ang II in Rat Offspring

**Figure 1:**
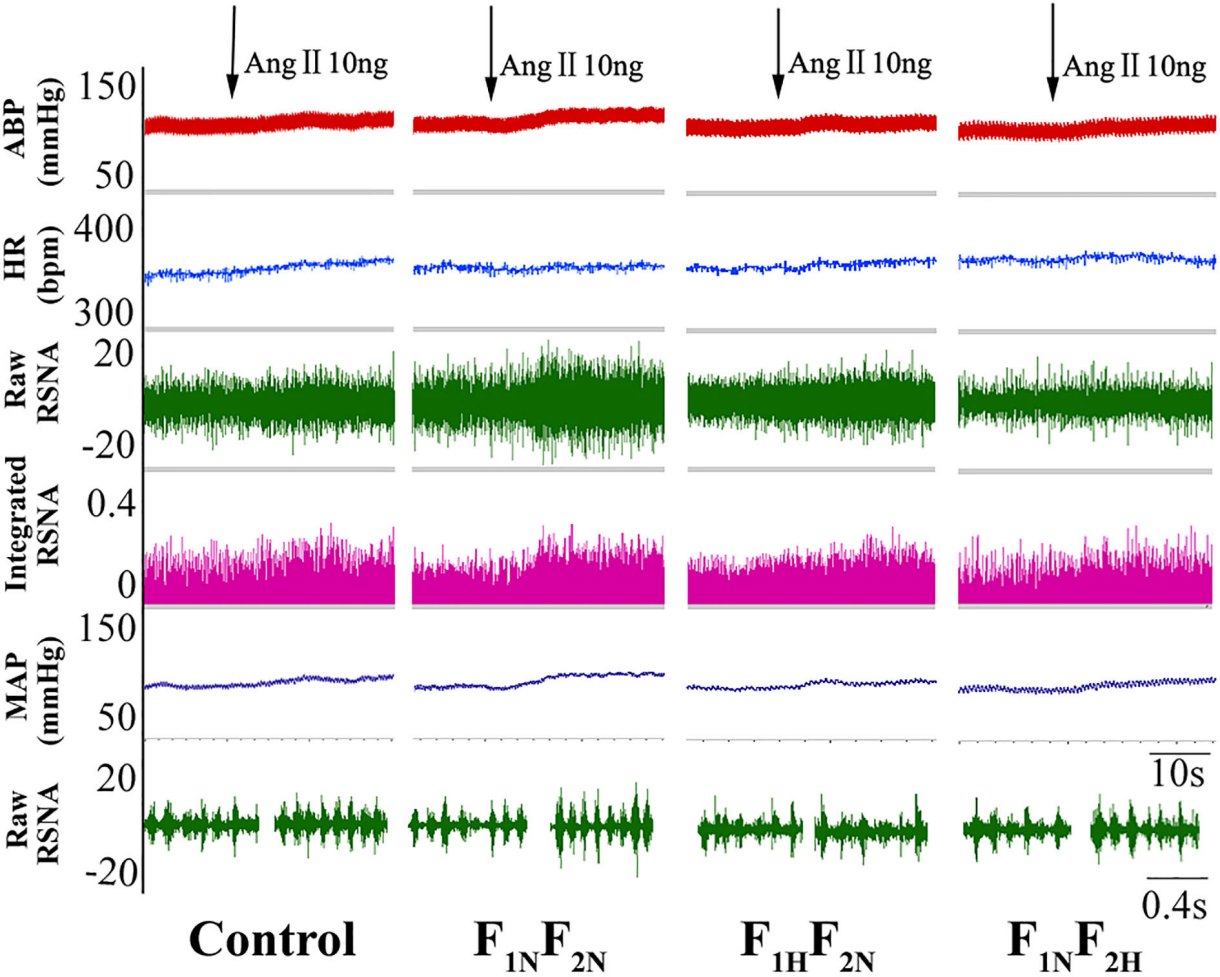
A raw trace recordings showing microinjection of Ang II (10 ng, 5 μL) into the lateral ventricles (LVs) of rats increased mean aterial pressure (MAP), HR, and renal sympathetic nerve activity (RSNA) in male offspring from the control, F_1N_F_2N_ (offspring of renovascular hypertension [RVH] rats), F_1H_F_2N_ (RVH rats treated with NaHS, offspring without NaHS), and F_1N_F_2H_ (maternal RVH rats treated without NaHS and offspring with NaHS after weaning) groups (*n* = 8).

**Figure 2:**
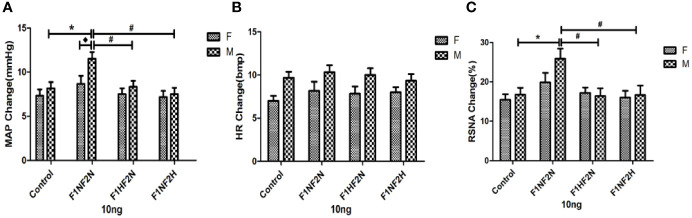
Microinjection of Ang II 10 ng into the LVs in both male and female offspring. Summary data showing the peak effect of Ang II (10 ng, 5 μL) microinjected into LVs on MAP **(A)**, HR **(B)**, and RSNA **(C)**. Data are mean ± SE (*n* = 8). *P < 0.05 compared with control, ^#^*P* < 0.05 compared with F_1N_F_2N_, and ^◆^*P* <0.05 male F_1N_F_2N_ compared with female F_1N_F_2N_.

Intracerebroventricular infusion of 10 ng of Ang II increased MAP, HR, and RSNA in both male and female offspring. **Figure 1** shows an original trace of the sympathetic response to the lower dose of Ang II (10 ng, 5 μL) with intracerebroventricular injection in male offspring. Sympathoexcitatory responses to intracerebroventricular injection of 10 ng of Ang II began within 1 min, reached a plateau within 3 to 5 min, and returned to the normal steady state within 10 min. **Figures 2A–C** show summary data in male and female offspring. Changes in MAP and RSNA were higher in the F_1N_F_2N_ than control group among male offspring (ΔMAP: 11.5 ± 0.76 vs. 8.17 ± 0.70 mmHg; ΔRSNA: 25.90 ± 2.53% vs. 16.78 ± 1.70%; *P*<0.05). As compared with the F_1N_F_2N_ group, in the F_1H_F_2N_ and F_1N_F_2H_ groups, the above effects were significantly attenuated (F_1H_F_2N_:ΔMAP, 8.33 ± 0.67 mmHg; ΔRSNA, 16.42 ± 1.98%; F_1N_F_2H_:ΔMAP, 7.50 ± 0.72 mmHg; ΔRSNA, 16.68 ± 2.41%; *P*<0.05). MAP, HR, and RSNA did not differ between female groups. However, the sympathetic responses change to lower dose of Ang II (10 ng, 5 μL) was higher in male than female RVH offspring (ΔMAP, 11.5 ± 0.76 vs. 8.67 ± 0.92 mmHg; *P*<0.05), with no sex differences in control offspring. **Table 2** showed the basal blood pressure and HR in offspring responsed to intracerebroventricular injection of 10 ng of Ang II. Sympathoexcitatory effects were similar with **Figures 2A–C**.

**Table 2:** Basal MAP and HR were measured under anaesthesia with a pressure transducer through a catheter placed in the left femoral artery.

Parameter				Control	F_1N_F_2N_	F_1H_F_2N_	F_1N_F_2H_
MAP(mmHg)	Ang II 10 ng	M	Baseline	98.3 ± 2.6	109.8 ± 2.7	99.5 ± 3.1	101.8 ± 2.8
			Ang II10 ng	106.5 ± 2.5	121.3 ± 2.9*	107.8 ± 2.8^#^	109.3 ± 2.5^#^
		F	Baseline	96.7 ± 2.8	106 ± 2.3	101.8 ± 1.6	98.8 ± 2.5
			Ang II10 ng	104 ± 3.2	114.7 ± 3	109.3 ± 1.5	106 ± 2.2
	Ang II 30 ng	M	Baseline	98 ± 3.2	110 ± 3	102.7 ± 2.6	101.5 ± 2.8
			Ang II30 ng	108.2 ± 3.3	123.3 ± 2.9*	111.5 ± 2.1^#^	110.2 ± 3^#^
		F	Baseline	98.3 ± 2.9	108.7 ± 2.5	99.2 ± 2.4	97.8 ± 2.8
			Ang II30 ng	106.3 ± 2.7	117.8 ± 2.7*	106.8 ± 2.4^#^	105 ± 2.7^#^
HR(bpm)	Ang II 10 ng	M	Baseline	341.7 ± 6.3	352 ± 8.4	346 ± 7.8	343.2 ± 6.2
			Ang II10 ng	351.3 ± 7	362.3 ± 8.8	356 ± 8	352.5 ± 5.8
		F	Baseline	342.8 ± 7.2	354.5 ± 5.6	349 ± 6.7	338.3 ± 8.9
			Ang II10 ng	349.8 ± 6.8	362.7 ± 5.2	356.8 ± 6.7	346.3 ± 9
	Ang II 30 ng	M	Baseline	344.7 ± 6.2	358.5 ± 7.2	345.5 ± 7.7	348.2 ± 6.1
			Ang II30 ng	354.8 ± 6.4	370.8 ± 6.7	355.7 ± 7.4	357.8 ± 6.4
		F	Baseline	349 ± 7.7	259.2 ± 7.2	353.2 ± 4.2	351.3 ± 5.3
			Ang II30 ng	353 ± 5.9	365.3 ± 6.9	361.3 ± 5.1	359.5 ± 5.2

Date are showed as mean ± SE (n = 8).*P < 0.05 compared with control group treatment with AngⅡ and ^#^P < 0.05 compared with F_1N_F_2N_ group treatment with AngⅡ.

#### Sympathetic Response to High-Dose Ang II in Offspring

**Figure 3:**
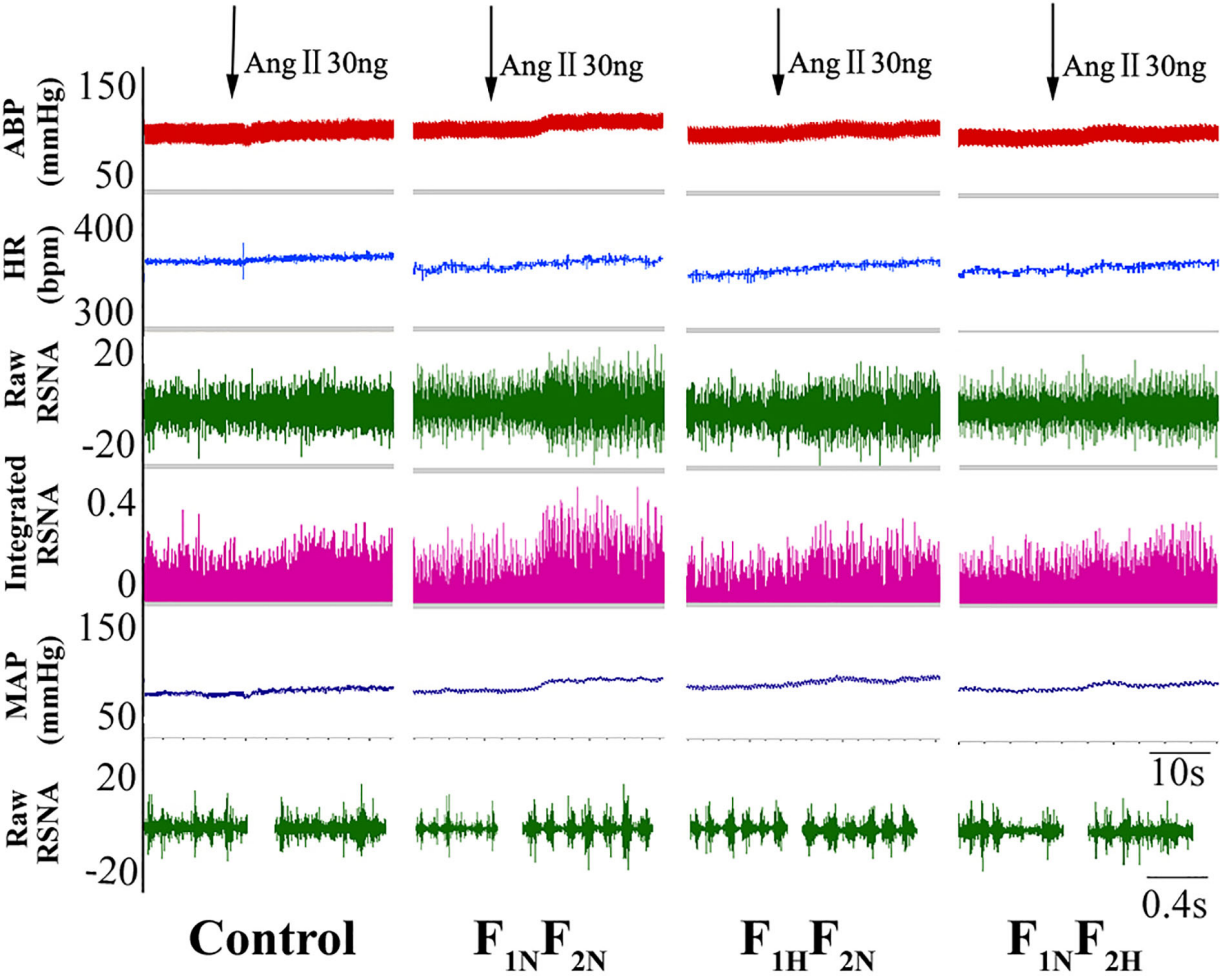
Original trace recordings showing microinjection of Ang II (30 ng, 5 μL) into LVs increased MAP, HR, and RSNA in female offspring from the control, F_1N_F_2N_ (offspring of RVH rats), F_1H_F_2N_ (RVH rats treated with NaHS, offspring without NaHS), and F_1N_F_2H_ (maternal RVH rats treated without NaHS and offspring with NaHS after weaning) groups (*n* = 8).

**Figure 4:**
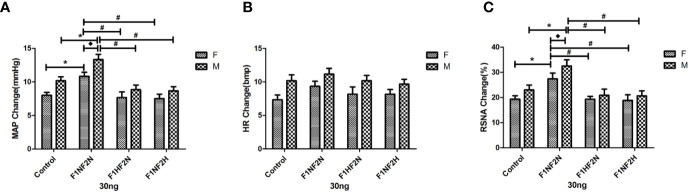
Microinjection of Ang II 30 ng into the LVs in male and female offspring. Summary data show the peak change of Ang II (30 ng, 5 μL) microinjected into LVs on MAP **(A)**, HR **(B)**, and RSNA **(C)**. Data are mean ± SE (*n* = 8). *P < 0.05 compared with control, ^#^*P* < 0.05 compared with F_1N_F_2N_, and ^◆^*P* < 0.05 male F_1N_F_2N_ compared with female F_1N_F_2N._

**Figure 3** shows the original tracing of the larger dose of Ang II (30 ng) inducing increased sympathetic outflow in female offspring. **Figures 4A–C** show summary data for sympathetic outflow change and that MAP and RSNA were higher in F_1N_F_2N_ than control female and male offspring (female offspring:ΔMAP, 10.83 ± 0.60 vs. 8.00 ± 0.45 mmHg; ΔRSNA, 27.38 ± 2.34% vs. 19.34 ± 1.39%, *P*<0.05; male offspring: ΔMAP, 13.33 ± 0.76 vs. 10.17 ± 0.60 mmHg;ΔRSNA, 32.50 ± 2.47% vs. 23.00 ± 1.93%; *P* < 0.05). As compared with the F_1N_F_2N_ group, F_1H_F_2N_ and F_1N_F_2H_ groups showed significantly decreased hypertension. HR showed no change at any dose of Ang II (**Figure 4**). Effects were similar with intracerebroventricular infusion of Ang II (3 and 10 ng) and were not dose-dependent. As compared with control offspring, RVH offspring showed sex differences. The sympathetic response to higher dose of Ang II (30 ng, 5 μL) was higher in male than female RVH offspring (ΔMAP, 13.33 ± 0.76 vs. 10.83 ± 0.60 mmHg; *P*<0.05; ΔRSNA:27.38 ± 2.34% vs. 32.5 ± 2.47%, *P* < 0.05). **Table 2** showed the basal blood pressure and HR in offspring responsed to intracerebroventricular injection of Ang II (30 ng).

### Protein Expression in NTS of Offspring

#### Protein Expression in NTS of Male Offspring

**Figure 5:**
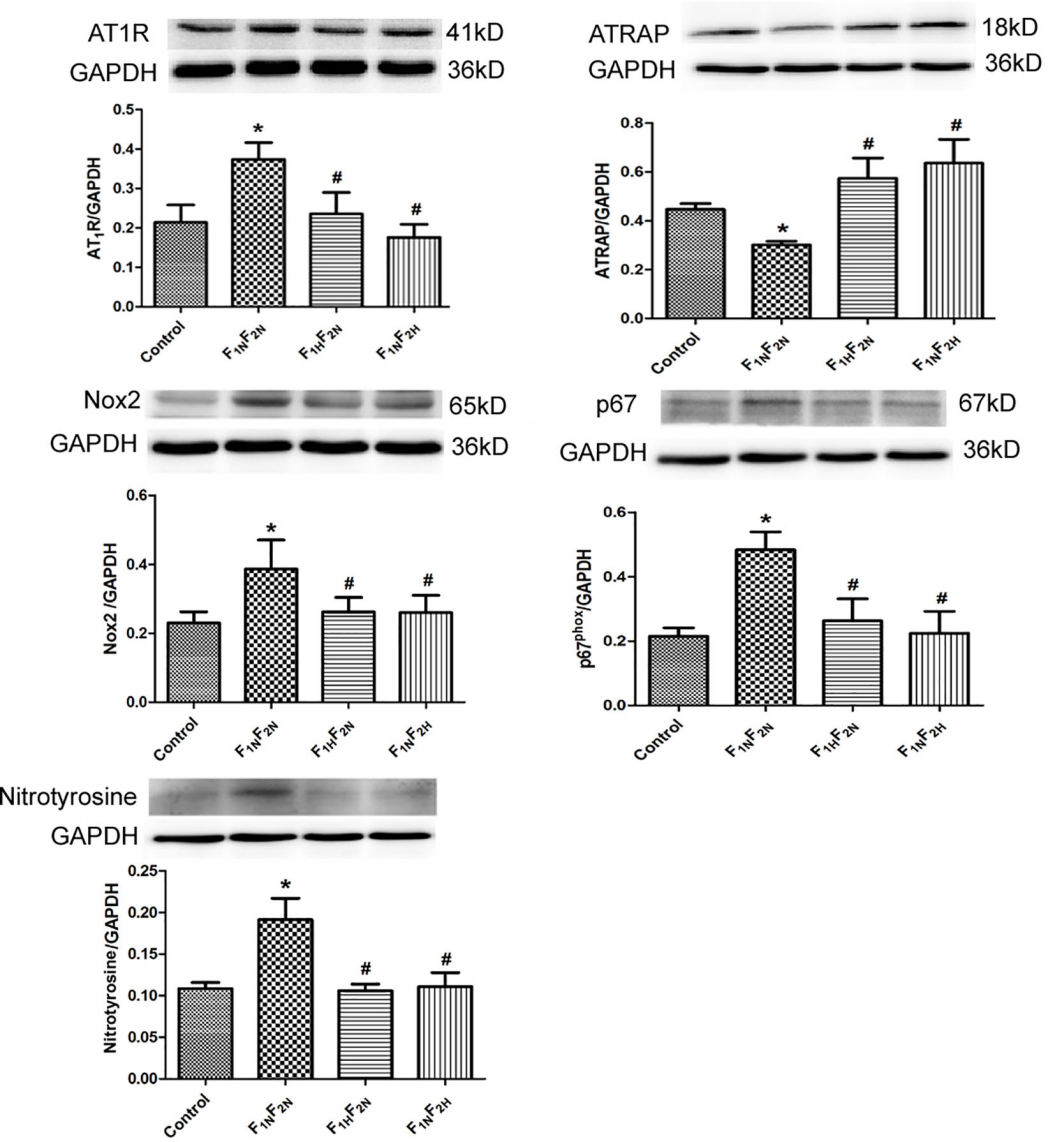
Western blot analysis of protein expression of AT1R, ATRAP, Nox2, p67phox, and nitrotyrosine in nucleus tractus solitarii (NTS) in male offspring from the control, F_1N_F_2N_ (offspring of RVH rats), F_1H_F_2N_ (RVH rats treated with NaHS, offspring without NaHS), and F_1N_F_2H_ (maternal RVH rats treated without NaHS and offspring with NaHS after weaning) groups. Data are mean ± SE (*n* = 12). *P < 0.05 compared with control and ^#^*P* < 0.05 compared with F_1N_F_2N_. GAPDH was used to normalize.

The protein expression of AT1R, Nox2, p67^phox^, and nitrotyrosine was significantly increased and that of ATRAP was decreased in the NTS in the F_1N_F_2N_ group; exogenous administration of hydrogen sulfide during prenatal or postnatal significantly reversed the above changes (**Figure 5**).

#### Protein Expression in NTS of Female Offspring

**Figure 6:**
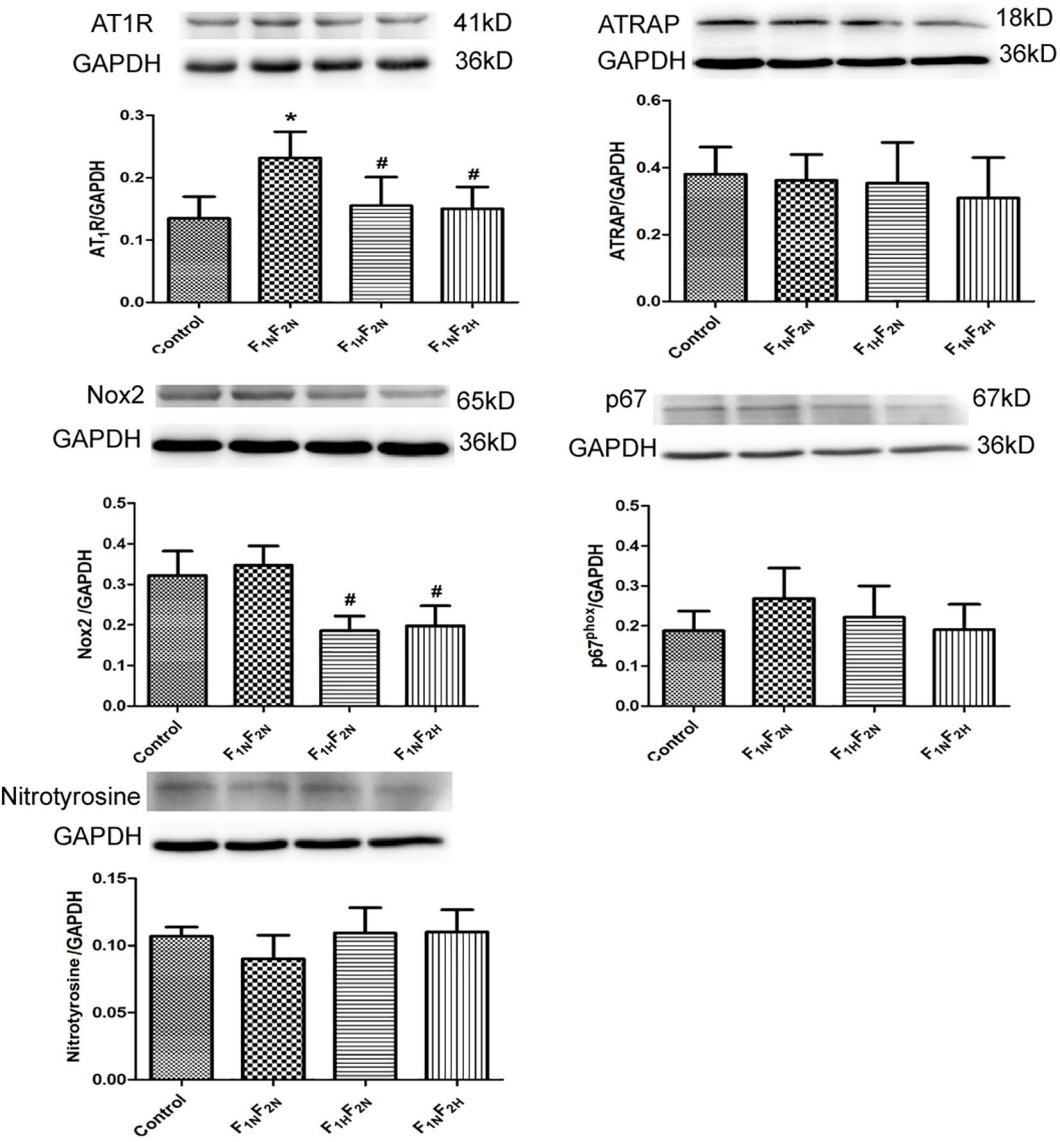
Western blot analysis of protein expression of AT1R, ATRAP, Nox2, p67phox, and nitrotyrosine in NTS in female offspring from the control, F_1N_F_2N_ (offspring of RVH rats), F_1H_F_2N_ (RVH rats treated with NaHS, offspring without NaHS), and F_1N_F_2H_ (maternal RVH rats treated without NaHS and offspring with NaHS after weaning) groups. Data are mean ± SE (*n* = 12). *P < 0.05 compared with control and ^#^*P* < 0.05 compared with F_1N_F_2N_. GAPDH was used to normalize.

As compared with controls, AT1R protein expression was significantly increased in F_1N_F_2N_ group, whereas the protein expression of ATRAP, Nox2, p67^phox^, and nitrotyrosine did not differ between groups. Prenatal or postnatal treatment with hydrogen sulfide significantly decreased the protein expression of AT1R in group (**Figure 6**).

## Discussion

**Figure 7:**
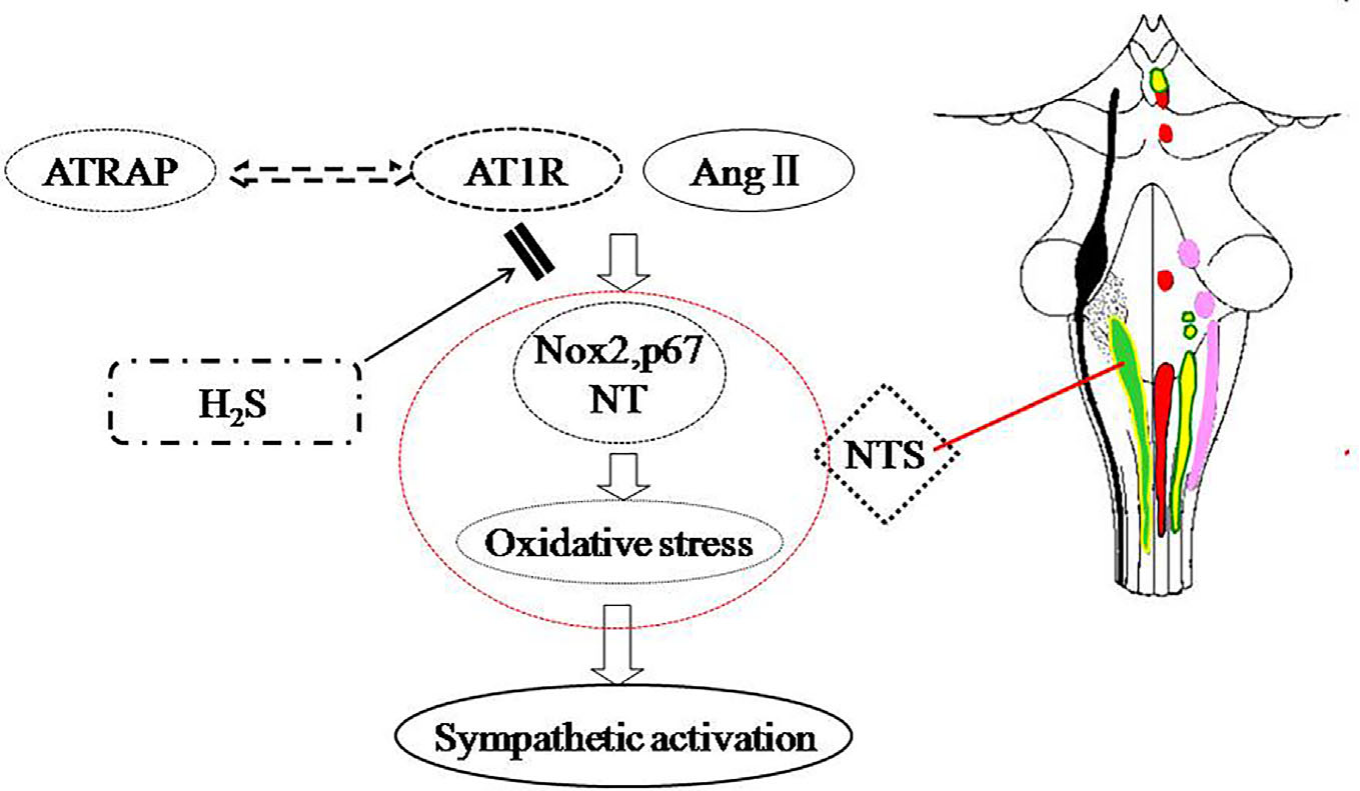
Schematic representation of the ameliorative effect of H_2_S on sympathetic activation in offspring of parental hypertension.

In this study, we have found that SBP in offspring of renovascular hypertensive significantly increased from 8 to 16 weeks; with intracerebroventricular infusion of Ang II, the parameters MAP, HR and RSNA were greater in offspring of parents with than without RVH. Offspring of normal rats showed no sex differences in BP or sympathetic outflow, but for RVH dams, BP and the sympathetic response to Ang II were both higher in male than female offspring; The expression of AT1 was increased and that of ATRAP decreased in NTS in offspring of RVH animals; prenatal or postnatal treatment with H_2_S could prevent the sympathetic activation in offspring (**Figure 7**).

Epidemiologic studies have shown that the maternal intrauterine environment is positively associated with BP in children (Longo et al., 2016). Maternal exposure to the nitric oxide (NO) synthase (NOS) inhibitor NG-nitro-l-arginine-methyl ester (L-NAME) during pregnancy induced hypertension in male offspring (Tain et al., 2016). Another research demonstrated that basal blood pressure in adult offspring of mothers with secondary renovascular hypertension was not increased. However, in our study, the baseline blood pressure in offspring of parents RVH animals was increased, which meant paternal or maternal status both have contribution to the offspring phenotype. Meanwhile, there is a study have demonstrated that sex difference in programmed hypertension is in male but not female adult offspring (Chiossi et al., 2016; Gray et al., 2016). To the contrary, our study have found that both male and female adult offspring of renovascular hypertensive rats have higher blood pressure, and the blood pressure was also higher in male offspring of renovascular hypertensive rats than that in female offspring. However, there was no sex difference in the offspring of normotensive rats.

Sex-specific differences in programmed disease have been well described in the literature. Denton et al. found that maternal secondary hypertension can “program” hypertension in female adult offspring. These results suggest sex-specific differences in sensitivity to the altered uteral environment (Denton et al., 2003). Consistent with these studies, our study demonstrated that parental secondary hypertension conferred sympathetic activation in adult offspring of both sexes. Furthermore, the sympathetic response to Ang II was greater in hypertensive male than female offspring. We found sex differences in the development of hypertension in offspring of RVH dams. Parental renovascular hypertension induced sympathetic activity in offspring sex-specifically. Our findings in conjunction with the above studies suggest that the offspring of hypertensive animals are prone to hypertension themselves.

Several mechanisms are considered involved in programmed hypertension, including NO deficiency, oxidative stress, epigenetic regulation, altered renin–angiotensin system and sodium transporters. The RAS plays a fundamental role in regulating sympathetic activity and BP control. Altered RAS in the brain and kidney may be an important mechanism for the fetal programming of hypertension (Guo et al., 2017; Xue et al., 2017). In our previous study, we found the AT1 receptor was epigenetically regulated in the kidney of maternal secondary hypertensive offspring (Guo et al., 2017). In the present study, intracerebroventricular infusion of Ang II (10 ng/5 µl) increased BP, HR, and RSNA in male hypertensive offspring and Ang II (30 ng/5 µl) in female hypertensive offspring.

ATRAP, a molecule that directly binds to the carboxyl-terminal domain of AT1R, could suppress the Ang II-AT1R–mediated pathological process (Lopez-Ilasaca et al., 2003). Also, increasing ATRAP level could ameliorate Ang II-induced hypertrophy in mice, with no effect on baseline cardiovascular function, including BP. Systemic ATRAP deficiency provoked vasoconstriction and increased sodium retention, exacerbating hypertension induced by chronic Ang II infusion. Increased ATRAP level inhibited salt-sensitive BP elevation (Wakui et al., 2015). All the above evidence indicate that activation of ATRAP plays an important role in inhibiting Ang II-dependent hypertension. Similarity, in our study, ATRAP was significantly decreased in the NTS of male offspring of hypertensive animals, and the sympathetic outflow was activated. However, ATRAP expression did not differ in female offspring, which suggests that ATRAP was not only involved in the development of hypertension in both male and female offspring but also was responsible for the sex differences in BP in these pups.

Oxidative stress is a key mediator in the pathogenesis of programmed hypertension. NADPH oxidases (Nox), a family of reactive oxygen species-producing enzymes, are a source of oxidative stress in diseases. Nox2 is the main ROS-producing enzyme producing superoxide and reducing NO bioavailability (Judkins et al., 2010). Preventing both the maternal and fetal adverse effects by inhibiting Nox2 may be an effective therapy for preeclampsia. Also, nicotine exposure during pregnancy leads to programming vascular dysfunction *via* a Nox2-dependent mechanism and increases the risk of hypertension in adult offspring (Xiao et al., 2011; Lim et al., 2015). One of the NADPH oxidase subunits, p67phox, also participates in fetal programming. In mothers with a low-protein diet, vascular remodeling was induced in offspring, which was attributed to increased p67phox level (Chisaka et al., 2016). Nitrotyrosine is considered a biomarker for endogenous level of peroxynitrite, and its level is correlated with elevated levels of other indices of oxidative stress. In line with previous studies, our study showed that Nox2, p67^phox^, and nitrotyrosine levels were increased in the NTS of male offspring of RVH animals. However, different from male offspring, female offspring showed no significant differences in Nox2, p67^phox^, and nitrotyrosine levels. Estrogen may play a protective role. Indeed, estrogen can combine with estrogen receptor and then inhibit the increase in reactive oxygen products and exert an antioxidant effect (Lagranha et al., 2010; Grundt et al., 2010; Siqueira et al., 2011).

H_2_S acts as a vasodilator and is widely expressed in mammals. Our previous study found that H_2_S could decrease BP, reduce the product of reactive oxygen species, and increase the methylation of AT1b receptor in kidney (Xue et al., 2015). Several studies revealed that H_2_S is involved in fetal programming. More importantly, reduced H_2_S levels in preeclampsia can lead to fetal growth restriction, whereas H_2_S treatment prevented pre-eclampsia and fetal growth restriction (Wang et al., 2013; Wang et al., 2014; Tai et al., 2016). Our data showed that prenatal or postnatal treatment with H_2_S could decrease sympathetic activation and basal blood pressure in hypertensive offspring, so H_2_S could modulate the balance between AT1R and ATRAP, reduce oxidative stress, and then improve the embryonic environment for offspring.

Our study has some limitations. We did not examine birth weight in offspring or the epigenetic mechanism of the AT1 receptor in the brain. Nevertheless, the data from the present study suggest the importance of sex differences in programmed hypertension, which is worthy of further investigation. Also, H_2_S may be a potential drug to prevent fetal programmed hypertension and can be a new method to treat hypertension.

## Data Availability Statement

The raw data supporting the conclusions of this article will be made available by the authors, without undue reservation.

## Ethics Statement

The animal study was reviewed and approved by Animal Care Committee of Hebei Medical University.

## Author Contributions

Conception and design: QG, XF, and YW. Conducted the research: XF, QG, and HX. Acquisition, analysis, and interpretation of data: XD and SJ. Manuscript preparation: QG, XF, and YW.

## Funding

This work is supported by Program for National Natural Science Foundation of China (NSFC) (31671185, 31701008, 31871154, and 91849120), Youth Top Talent Program Foundation, Hebei Province of China, the Province Natural Science Foundation of Hebei (No. H2020206307), and China Scholarship Foundation (CSC.201808130056).

## Conflict of Interest

The authors declare that the research was conducted in the absence of any commercial or financial relationships that could be construed as a potential conflict of interest.
